# Construct validity of the Charitable Food Nutrition Index

**DOI:** 10.1016/j.pmedr.2023.102515

**Published:** 2023-11-14

**Authors:** Maria F. Gombi-Vaca, Ran Xu, Marlene B. Schwartz, Caitlin E. Caspi

**Affiliations:** a Rudd Center for Food Policy and Health, University of Connecticut, One Constitution Plaza, Suite 600, Hartford, CT 06103, United States; b Department of Allied Health Sciences, University of Connecticut, 358 Mansfield Rd, Unit 1101, Storrs, CT 06269, United States; c Department of Human Development and Family Sciences, University of Connecticut, 348 Mansfield Road, Unit 1058, Storrs, CT 06269, United States

**Keywords:** Charitable food system, Food pantry, Nutrition guidelines, Construct validity, Implementation, Intervention

## Abstract

**Background:**

Food pantries are an important source of food for those facing food insecurity. The Charitable Food Nutrition Index (CFNI) was developed for research and practice to measure the nutritional quality of assortments of foods in this setting.

**Objective:**

The study assessed the construct validity of the CFNI using secondary data from a group-randomized food pantry intervention in Minnesota.

**Methods:**

The CFNI was calculated for each client cart post-intervention (n = 187; 85 intervention, 102 control). CFNI scores were based on the proportion of items in each client cart ranked “green,” “yellow,” or “red” using the Healthy Eating Research Nutrition Guidelines for the Charitable Food System. An implementation score assessing intervention fidelity was measured for each pantry (n = 11; 5 intervention, 6 control) based on the four intervention subcomponents: aesthetics/use of space; healthy food prominence and appeal; unhealthy food de-emphasis; and stocking standards. Mixed linear models were used to test whether: (a) client carts from pantries in the intervention condition had higher CFNI scores than those in the control condition, and (b) higher implementation scores were associated with higher CFNI scores.

**Results:**

In adjusted models, clients from intervention group pantries had higher CFNI scores, reflecting a healthier assortment of foods compared with clients from control group pantries (p = 0.022). CFNI scores were positively associated with greater fidelity to the intervention (p = 0.020).

**Conclusions:**

The CFNI was sensitive enough to detect the effects of the intervention in the expected directions. These findings support its construct validity and utility as a measure in the charitable food system.

## Highlights

• The Charitable Food Nutrition Index (CFNI) measures nutritional quality of foods.
• The CFNI is sensitive enough to detect intervention effects.
• Findings support the construct validity of the CFNI.

## 1 Introduction

Each year in the U.S., millions of food insecure households rely on the charitable food system to meet their food needs (Coleman-Jensen et al., 2022). Research suggests that many clients rely on food pantries for a considerable amount of their food and for long periods of time (Caspi et al., 2019, Liu et al., 2020a, Wright et al., 2018, Wright et al., 2020). Historically, the charitable food system measured its success by the number of pounds of food distributed (Martin, 2021). The priorities have changed, however, due to growing recognition that people who use the charitable food system are at higher risk of poor diet quality (Caspi et al., 2021, Liu et al., 2020b, Liu et al., 2019, Wright et al., 2018) and chronic diseases such as obesity and diabetes (Franklin et al., 2012, Gregory and Coleman-Jensen, 2017, Gundersen and Ziliak, 2015, Laraia, 2013, Leung et al., 2020, Seligman et al., 2010, Seligman et al., 2007). Over the past several years, there have been expanded efforts to develop interventions to improve the nutritional quality of food in food pantries with the goal of promoting healthier food consumption among clients (Byker Shanks, 2017, Caspi et al., 2019, Coombs et al., 2020, Follett et al., 2022, Gibson et al., 2022, Quinn et al., 2021, Schwartz et al., 2020).

The recent emphasis on healthy food in the charitable food system has exposed the challenges of measuring the nutritional quality of food procured and distributed in the charitable food system. To address the unique needs in this setting, the Healthy Eating Research (HER), a national program of the Robert Wood Johnson Foundation, established an expert panel to develop the HER Nutrition Guidelines for the Charitable Food System (Schwartz et al., 2020). These Guidelines have since been recognized by Feeding America, the largest network of food banks in the U.S. Feeding America has also supported the implementation of the guidelines within the network through grants and a toolkit in 2021 (Feeding America, 2021). The HER Guidelines outline a process for sorting foods into eleven categories (fruits and vegetables; grains; protein; dairy; non-dairy alternatives; beverages; mixed dishes; processed and packaged snacks; desserts; condiments and cooking staples; or other miscellaneous products) and ranking them in a tiered system: green (choose often), yellow (choose sometimes), red (choose rarely). Rankings are based on foods’ nutrient profiles for saturated fat, sodium, and added sugars, and the presence of whole grains.

The HER Guidelines are used to rank individual foods; however, characterizing an *assortment* of foods requires three numbers (e.g., the percentage of green, yellow, and red foods in a shopping cart or food pantry). In response, the Charitable Food Nutrition Index (CFNI) was developed to convert these three values into one continuous score of overall nutritional quality that can be used to assess any assortment of foods (Gombi-Vaca et al., 2022). The highest score for the CFNI possible (1 0 0) would be obtained for an assortment of foods that has 100 % green foods, and lowest value possible (0) would be obtained for an assortment of foods that has 100 % red foods. The CFNI has a moderate-to-strong correlation of 0.585 (p < 0.001) with the Healthy Eating Index (HEI) (Gombi-Vaca et al., 2022), which is a valid measure of the alignment of a set of foods with Dietary Guidelines for Americans (Kirkpatrick et al., 2018, Reedy et al., 2018), and is more feasible for both charitable food agencies and researchers to calculate.

As the nutritional quality assessment of foods using tier-ranked approach is increasingly being promoted in the charitable food system (UNC. Center for Health Promotion and Disease Prevention, 2023), it is important to build the understanding of the utility of measures like the CFNI in research and practice. Testing the construct validity of the CFNI can determine whether it is a useful instrument for capturing improvements in the charitable food system. In other words, do CFNI scores “behave” as expected (DeVellis, 2003), with higher scores reflecting a higher quality nutritional environment and lower scores reflecting a lower quality nutritional environment? One way to test construct validity is to determine whether the CFNI detects the effects of an environmental intervention designed to improve the nutritional quality of client food selections in food pantries.

In the current study, we conducted a secondary analysis of the group-randomized SuperShelf intervention, a community-led initiative in choice-based food pantries (i.e., where clients can select their own foods) that centered on environmental changes and behavioral economics strategies. Behavioral economics strategies aim to make the healthy choice the easier choice for clients (Anderson et al., 2021, Caspi et al., 2019, Coombs et al., 2020, McKee et al., 2021, Stein et al., 2019, Wilson et al., 2016). We assessed the construct validity of CFNI by testing (a) whether the food selected by clients in the intervention group had higher CFNI scores compared with the food selected by clients in the control group, and (b) whether the strength of intervention implementation was associated with CFNI scores.

## 2 Methods

This study is a secondary analysis using data collected in a group-randomized intervention study of food pantries in Minnesota (Caspi et al., 2023, SuperShelf: Evaluation, n.d.). The pre-intervention (baseline) data were collected in two waves in 2018 and 2019, and the post-intervention (follow-up) data were collected at each pantry one year after the pre-intervention measure. Data from 11 sites that completed both pre- and post-intervention measures were included in the present analyses. All pre- and post-intervention data collection occurred during the same two-week period in each food pantry at each time point.

### 2.1 Intervention

SuperShelf was a behavioral economics intervention that aimed to transform food pantries (known as “food shelves” in Minnesota) by creating welcoming environments and greater access to a variety of appealing, healthy foods (Caspi et al., 2023, SuperShelf, n.d.). The intervention components were community-led and supported by early evidence that behavioral economics is an effective strategy to increase healthy food selection (Caspi et al., 2019, Wilson et al., 2017). The intervention occurred in two phases over a period of several months. The first part of the intervention was designed to increase the supply of healthy options, because adequate supply of healthy food is a prerequisite for promoting healthy foods to clients. In the second phase of the intervention, the food pantry underwent an environment transformation which implemented behavioral economics strategies to improve the shopping experience for clients. The food pantry layout was changed, and food was arranged into major food group categories, as is found in a grocery store. The layout within the pantry and on the shelf was designed to “nudge” healthy behaviors by, for example, placing whole grain items at eye level or displaying fruits and vegetables in prominent baskets. Finally, appealing signage was used to promote healthy foods and label food groups. Although these strategies were not selected based on the HER Guidelines, they align with the purpose of the intervention of promoting healthier options (green and yellow foods) and de-emphasizing less healthy foods (red foods).

Participating pantries were located in Minnesota and were required to have “full client-choice”, meaning a food distribution process in which food from all food groups was displayed on shelves for clients to select or decline (Caspi et al., 2023, Caspi et al., 2022, Caspi et al., 2021). The selected pantries were placed into pairs prior to randomization based on region and urbanicity using Rural-Urban Commuting Area (RUCA) Codes 1–10 (USDA ERS - Rural-Urban Commuting Area Codes, n.d.). Randomization was conducted using a virtual coinflip generator. Control food pantries were offered a delayed intervention opportunity after follow-up measures were completed.

The intervention study used a repeated cross-sectional design to evaluate the nutritional quality of the food selected by clients, so that one sample of clients was recruited at baseline and a second sample at post-intervention. All clients visiting the pantry on the day of data collection were approached and screened by the research team after they had selected their food. Participants were eligible to participate if they were 18 years old, spoke English, Spanish, or Somali, were mentally capable of consent and participation, and had access to a phone.

After obtaining informed consent, participants completed a survey with questions about food pantry usage and demographics. While clients completed the survey, the research team recorded the foods selected by each client at their visit.

### 2.2 Client measures and food selection

The following client demographics variables were collected in the survey: self-classified gender; age; self-classified racial background; self-classified ethnicity; education; and household size. For the current analysis, participant age was categorized into age groups and the dimensions of race and ethnicity were combined into one race/ethnicity variable. Clients were also asked to estimate the proportion of their food that came from a pantry in the prior 6 months and how frequently they visited the food pantry in the last 12 months.

Research staff photographed each food item selected with a study-specific iPod touch device. Picture of the packaged items captured the product name, brand, size, quantity, and special nutritional notes on the label (e.g., reduced sodium, reduced fat). Pictures of non-packaged items (e.g., produce) were photographed on a scale that displayed the weight. Following the pantry visit, data from the pictures were entered into an Excel database, which was then entered into Nutrient Data Systems for Research (NDSR). NDSR is a software application that allows for direct entry of dietary data in a standardized fashion (NDSR Software, n.d). The Nutrition Coordinating Center Food and Nutrient Database (Sievert et al., 1989) generated values for saturated fat, sugar, and sodium needed to rank each food item using HER Guidelines.

### 2.3 HER guidelines measures and CFNI

Each food item in the client carts (n = 16,404 total items) was ranked according to HER Guidelines (Schwartz et al., 2020). First, items were designated into their food category, and then assigned green (choose often), yellow (choose sometimes), red (choose rarely), or not ranked based on the food category and amount of saturated fat, sodium, and added sugar per serving in the item (Schwartz et al., 2020).

For this analysis, not ranked foods (n = 1,353, 8.2 %) were excluded. Not ranked foods included condiments (e.g., salad dressing, soy sauce), cooking staples (e.g., flour, oil), and miscellaneous foods (e.g., baby food, nutritional supplements). According to HER Guidelines, these foods are not scored because they are not typically consumed on their own or in large quantities (Schwartz et al., 2020). They may also be paired with more nutritious ingredients and used in the preparation of healthy meals. Miscellaneous foods are not ranked because they are intended for special populations with unique nutritional needs.

For each client cart (n = 399; 212 at pre-intervention and 187 at post-intervention) in the 11 food pantries, the percentage by weight of green, yellow, and red foods were generated by dividing the number of pounds of food from each color category by the total number in pounds of food in the cart. Next, the CFNI for each client cart was calculated using a formula that combines the percentage by weight of green, yellow, and red foods and weights for each percentage. These weights were empirically generated from statistical learning methods described elsewehere (Gombi-Vaca et al., 2022). The formula for the CFNI is the following (Gombi-Vaca et al., 2022, Rudd Center for Food Policy and Health, 2022):

$$\begin{array}{cc} \mathrm{CFNI} & =(\left((0.7773\times Percentage\;Green)+(0.5923\times Percentage\;Yellow\right)\\ & \;+(0.3753\times Percentage\;Red)-37.53)/40.20)\times 100. \end{array}$$

### 2.4 Food pantry measures and implementation scores

At pre- and post-intervention, a manager at each food pantry completed a survey about the number of pounds of food distributed monthly and the number of freezers and coolers. Food pantry urban/rural status was assigned based on RUCA code classifications (USDA ERS - Rural-Urban Commuting Area Codes, n.d.).

Fidelity to the intervention was determined by measuring the extent to which intervention components were implemented as intended (Caspi et al., 2022). A research team member walked through each pantry with a checklist at pre- and post-intervention to assess the pantry environment. This assessment was also done in control condition food pantries to capture secular changes occurring in the broader charitable food sector. The implementation score ranged from 0 to 100 and was based on the sum of four intervention components: 1) aesthetics/use of space (28 points); 2) healthy food prominence and appeal (22 points); 3) unhealthy food de-emphasis (21 points); and 4) stocking standards (29 points). The fidelity scores were coded so that higher scores represent higher fidelity. The fidelity scoring tool and the questions asked representing each intervention component have been detailed elsewhere (Caspi et al., 2022, Caspi et al., 2019).

### 2.5 Data analysis

The main analysis was based on measures from 187 client carts and 11 food pantry implementation checklists collected in the post-intervention period. Pre-intervention client cart measures (n = 212) were used to calculate pre-intervention food pantry CFNI scores, which were used as covariates in the adjusted models, along with pre-intervention implementation scores (total and subcomponents) from the 11 food pantries.

The first aim was to test whether the food selected by clients in the intervention group had a higher nutritional quality than the food selected by clients in the control group, using CFNI measures. We used mixed linear models that includes a random effect term for food pantry to account for clients clustered within each food pantry. The dependent variable was the client cart CFNI score, and the independent variable was the intervention arm (intervention or control). Fully adjusted models included food pantry pre-intervention CFNI to account for differences in the food pantry environment at baseline. Models also included client demographics (gender, age group, race/ethnicity, education, and household size), client food pantry usage (proportion of their food obtained from pantry in the last 6 months, and food pantry visit frequency in the last 12 months), and food pantry characteristics at post-intervention (monthly pounds of food distributed, number of freezers and coolers, and pantry location).

For the second aim, mixed linear regression models that accounted for clients clustered within pantries were used to test the association between total and subcomponent implementation scores and CFNI scores. We fit a model for total implementation score and a model for each of the subcomponents. The dependent variable was client cart CFNI score and the independent variable was the implementation total score or the subcomponent score. Fully adjusted models included pre-intervention food pantry CFNI and pre-intervention food pantry implementation score (total or each of the four subcomponents). Models were adjusted by the same client and food pantry characteristics detailed above.

Human subject procedures were in accordance with the ethical standards of the institution and with the 1964 Helsinki declaration and its later amendments. The study was approved as protocol 1612S02201 at the University of Minnesota and H20-0076 at the University of Connecticut. Written informed consent was obtained from all individual participants included in the study.

## 3 Results

Client (n = 187) and food pantry (n = 11) characteristics by intervention arm are shown in Table 1. On average, for the whole sample, client carts were comprised of 49.9 % (SD = 13.2) green foods by weight, 27.0 % (SD = 10.6) yellow foods by weight, and 23.1 % (SD = 10.9) red foods by weight. CFNI scores were on average 65.5 (SD = 10.7) and 63.7 (SD = 10.9) for the intervention pantries and for the control pantries, respectively.

**Table 1:** Distribution of client and food pantry characteristics by intervention arm (Minnesota, 2019–2020).

	**Intervention** **(n = 85)**		**Control** **(n = 102)**	
**Client characteristics**	*n*	*(%)*	*n*	*(%)*
**Gender**				
Female	49	(58.3)	63	(61.8)
Male	34	(40.5)	39	(38.2)
Transgender	1	(1.2)	0	(0)
**Age group**				
18 to 44 years old	36	(42.9)	44	(44.0)
45 to 64 years old	42	(50.0)	44	(44.0)
≥ 65 years old	6	(7.1)	12	(12.0)
**Race/Ethnicity**				
Hispanic-Latino/a	13	(15.7)	10	(10.3)
Non-Hispanic White	46	(55.4)	53	(54.6)
Non-Hispanic Black	10	(12.1)	17	(17.5)
Non-Hispanic Native American	4	(4.8)	4	(4.1)
More than one race or Other^a^	10	(12.1)	13	(13.4)
**Education**				
Less than high school	8	(9.6)	6	(6.2)
High school or graduate equivalency degree	33	(39.8)	36	(37.1)
Some college/associates/vocational- technical degree	33	(39.8)	46	(47.4)
Four-year college degree or higher	9	(10.8)	9	(9.3)
**Proportion of food obtained from pantry in the last 6 months**				
Half or more	43	(51.8)	53	(52.5)
Less than half	40	(48.2)	48	(47.5)
**Food pantry visit frequency in the last 12 months**				
Once a month or more	60	(72.3)	75	(73.5)
Less than once a month	23	(27.7)	27	(26.5)
**Household size**, *n, Mean (SD)*	81	3.1 (2.0)	97	2.7 (1.9)

	**Intervention** **(n = 5)**		**Control** **(n = 6)**	
**Food pantry characteristics**	*n*	*(%)*	*n*	*(%)*

**Pantry number of freezers/coolers**				
More than 6	3	(60)	2	(33.3)
6 or fewer	2	(40)	4	(66.7)
**Pantry location**				
Urban	3	(60)	3	(50)
Rural	2	(40)	3	(50)
**Pantry pounds of food served per month**, *Mean (SD)*	38,135	(35,163)	35,398	(15,810)

a Participants that selected more than one of the possible responses to the self-classified racial background question, self-classified as “Native American” and “White or Caucasian”; “Native American”, “Black, African American” and “White”; “Black, African American” and “White”; or a unique participant write-in response.

The unadjusted analysis comparing mean client cart CFNI scores between intervention and control group found no statistically significant difference (*β* = 1.9; SE = 3.2; p = 0.556). In the fully adjusted models, client carts in the intervention group had a higher mean CFNI score than those in the control group (*β* = 3.7; SE = 1.6; p = 0.022).

The findings for the second aim testing the association between total and subcomponent implementation scores and client cart CFNI scores are displayed in Table 2. We found no statistically significant association between total and subcomponent implementation scores and CFNI in the unadjusted models. In the fully adjusted models, we found that CFNI scores were positively associated with total implementation score (*β* = 0.10; p = 0.020). We also found CFNI scores were associated with three of the four subcomponents: aesthetics/use of space (*β* = 0.29; p = 0.011), healthy food prominence and appeal (*β* = 0.54; p = 0.011), and unhealthy food placement and competition (*β* = 0.59; p = 0.004). Stocking standards subcomponent scores were not statistically significantly associated with CFNI scores (*β* = 0.28; p = 0.271).

**Table 2:** Mean implementation scores (total and subcomponents) and results from unadjusted and adjusted models testing the association between total and subcomponent implementation scores and client cart CFNI scores (Minnesota, 2019–2020).

	**Mean implementation score**		**Effect estimates for CFNI scores**					
	**Post-Intervention**		**Unadjusted models (n = 187)**			**Adjusted models**^a^**(n = 165)**^b^		
	*Mean*	*SD*	*β*	*SE*	*p-value*	*β*	*SE*	*p-value*
**Total and subcomponent implementation scores (maximum score possible)**								
Total (100 points)	70.4	20.6	0.07	0.08	0.328	0.10	0.04	0.020
Aesthetics/use of space (28 points)	18.3	7.2	0.20	0.22	0.359	0.29	0.11	0.011
Healthy food prominence and appeal (22 points)	14.5	4.6	0.31	0.34	0.355	0.54	0.21	0.011
Unhealthy food de-emphasis (21 points)	15.2	5.2	0.33	0.29	0.269	0.59	0.21	0.004
Stocking standards (29 points)	22.4	5.7	0.17	0.28	0.547	0.28	0.25	0.271

CFNI: Charitable Food Nutrition Index.

a Adjusted models included pre-intervention food pantry CFNI scores, pre-intervention subcomponent or total implementation scores, client demographics (gender, age group, race/ethnicity, education, and household size), client food pantry usage (proportion of food obtained from pantry in the last 6 months and food pantry visit frequency in the last 12 months), and food pantry characteristics (monthly pounds of food distributed, number of freezers and coolers, and pantry location).

b Sample size in the adjusted models is n = 165 due to missing data in covariates.

## 4 Discussion

In this analysis, we found that the Charitable Food Nutrition Index (CFNI) detected the effects of the SuperShelf intervention in the expected direction. Using the CFNI, the foods selected by clients in the intervention group had a higher nutritional quality compared with the foods selected by clients in the control group. We also found that, as expected, the strength of intervention implementation was associated with higher client cart CFNI scores. These findings build the evidence base for the CFNI as a valid measure for research and practice in the charitable food system.

The SuperShelf intervention study offered a reasonable setting in which to test whether the CFNI measure would behave in a way that was consistent with the theoretical hypothesis. This intervention implemented choice architecture to encourage the selection of foods from healthier categories, including fruits and vegetables and whole grains, while discouraging the selection of less healthier food categories, including mixed dishes, snacks, and desserts. In the SuperShelf evaluation study, the average improvement in implementation score was 33.2 in the intervention group, whereas control food pantry implementation scores decreased by an average of 3.8 points (Caspi et al., 2022). Given the demonstrated improvements in food pantry environment that resulted from the intervention (SuperShelf Gallery, n.d), we expected to observe corresponding changes in our measure of nutritional quality of client food choices. This study found that increase in SuperShelf implementation score was positively associated with increase in CFNI scores, and that client carts in the intervention pantries had higher CFNI scores than client carts in the control pantries. This suggests the CFNI was effective in measuring the modest improvement in the nutritional quality of food selected by clients in this particular environmental intervention.

However, it is notable that SuperShelf did not aim to implement the specific color-ranking system outlined in HER Guidelines. HER Guidelines would suggest applying a red label to foods like mixed dishes, snacks, and desserts, which tend to be high in saturated fat, added sugar, and sodium in order to de-emphasized them to clients; in the SuperShelf intervention, these foods were not labeled red, but they were placed at the end of the route that clients followed to select their food in order to de-emphasize them. It is possible that CFNI scores would be even better able to detect intervention effects when using HER Guidelines-specific tools and materials in the intervention, such as those offered in the Supporting Wellness at Pantries SNAP-Ed toolkit, a set of educational and practical resources designed to help implement HER Guidelines nutrition ranking in the charitable food system (UNC. Center for Health Promotion and Disease Prevention, 2023).

The original study was designed to assess intervention effects in the nutritional quality of client carts using the HEI as an outcome (Caspi et al., 2023). Although the HEI is a validated measure widely used to assess nutritional quality, there are key differences between the CFNI and HEI that may make the CFNI a more appropriate measure of nutritional quality in the charitable food setting. The HEI is not a measure that is commonly used to monitor the nutritional quality of the food pantry outside of research evaluations because it takes considerable resources to use, for example, it requires linking to relevant databases with information on nutrients and expertise in specific analytical software and coding (USDA, 2023, USDA, n.d.). Moreover, HEI is derived from the sum of 13 subcomponents, not all of which might be equally addressable targets for client choice interventions. For example, dairy (a subcomponent of HEI) is frequently low in availability at food pantries (Simmet et al., 2017) and is among the most difficult to source and store in the chartable food setting (Feeding America, 2018).

Another key difference between HEI and the CFNI is the exclusion of not ranked foods in CFNI measures. Not ranked foods consist of condiments, cooking staples, and miscellaneous foods. Such foods might contribute to lower HEI scores if they contain large amounts of saturated fat, sodium, or added sugar; at the same time, they are expensive to buy (and so might be logical for food pantry clients to take) and may be used only sparingly in home meal preparation. Indeed, the SuperShelf intervention did not discourage the selection of condiments, cooking staples, and miscellaneous foods and placed these foods together in the middle of the shopping experience (e.g., after proteins and dairy, before processed foods). Excluding such foods in a nutritional quality score in this setting may be a better way of capturing more important targets of client behavior – such as the selection of fruits and vegetables.

This study has several limitations. As it is a secondary analysis of a SuperShelf intervention, the study did not have an *a priori* goal of observing changes in the CFNI. The original intervention study was powered to detect differences between groups using HEI as an outcome and not CFNI scores. Finally, while significant differences in the CFNI were observed, the magnitude of these differences cannot be generalized to other interventions. Interventions based on the HER Guidelines may be even better suited to validate the CFNI.

In future intervention studies targeting the healthfulness food in food pantries, the CFNI may be a useful continuous outcome measure. The CFNI is built on the HER Guidelines that were developed specifically for the charitable food system. Moreover, while it correlates with the HEI, it requires fewer resources to calculate for both researchers and charitable food agencies. The CFNI can be easily calculated with any set of data that has been tier-ranked using HER Guidelines. As HER Guidelines have been endorsed by Feeding America and are being encouraged in food banks and pantries in the U.S., new opportunities with strong experimental study designs are likely to emerge to evaluate nutritional changes in the rapidly changing charitable food sector. It may also be a useful tool for food pantries for day-to-day monitoring food supply or client selections. However, successful implementation of HER Guidelines to accurately calculate CFNI scores may be dependent on food banks and pantries capacity, or in extra resources and funding.

### 4.1 Conclusions

Growing evidence suggests that the CFNI is an appropriate outcome measure for use in intervention studies in the charitable food system. As a validated measure that is relatively simple to calculate, the CFNI can also be used by charitable food agencies to monitor and report the nutritional quality of their food over time or between sources. However, support to implement HER Guidelines is key to obtaining reliable CFNI measures. Nonetheless, results from the current study offer additional support for the validity and utility of the CFNI.

## Funding sources

This research project was supported by Healthy Eating Research, a program of the Robert Wood Johnson Foundation (Prime Award 76093, Subaward 2834066, PI C.C.). Data used in this study were based on a study supported by the National Institute of Heart, Lung, and Blood Institute of the National Institutes of Health (1R01HL136640, PI C.C.), and the National Center for Advancing Translational Sciences (UL1TR002494, PI C.C.) supported data management. The content is solely the responsibility of the authors and does not necessarily represent the official views of Healthy Eating Research/Robert Wood Johnson Foundation or the National Institutes of Health.

## Declaration of Competing Interest

The authors declare that they have no known competing financial interests or personal relationships that could have appeared to influence the work reported in this paper.

## Data Availability

Data will be made available on request.
